# Comparison of the Effectiveness of Traditional Motorized Traction and Non-surgical Spinal Decompression Therapy Added to Conventional Physiotherapy for Treatment of Chronic Low Back Pain

**DOI:** 10.7759/cureus.69610

**Published:** 2024-09-17

**Authors:** Sevda Adar, Onurhan Apaydın, Umit Dündar, Hasan Toktas, Hilal Yesil, Selma Eroglu, Nuran Eyvaz

**Affiliations:** 1 Physical Medicine and Rehabilitation, Afyonkarahisar Health Sciences University, Afyonkarahisar, TUR

**Keywords:** physiotherapy, decompression, traction, intervertebral disc, low back pain

## Abstract

Background: There are a limited number of studies comparing traditional motorized traction and non-surgical spinal decompression with other treatment options such as conventional motor traction in the treatment of low back pain caused by lumbar discopathy. This retrospective study aimed to compare the effectiveness of these treatments.

Methods: The retrospective data of patients diagnosed with lumbar discopathy who underwent physical therapy in our clinic were reviewed. Demographic data, duration of their symptoms, physical examination findings, lumbosacral magnetic resonance imaging (MRI) reports, method and duration of treatment, and visual analog scale (VAS) and Oswestry Disability Index (ODI) results were recorded.

Results: A total of 160 patients met the inclusion criteria. Their mean age was 44.6±12.4 (range 21-65) years, 57.5% (n=92) were female, and 42.5% (n=68) were male. There were no differences between the conventional physiotherapy, motorized traction, and spinal decompression groups in terms of age, duration of symptoms, and the number of sessions (p>0.05). In all three groups, the mean scores of VAS and ODI were significantly decreased in the pre-and post-treatment comparisons (p<0.005). The rates of change in VAS and ODI were higher in the traction group and spinal decompression group compared to the conventional treatment (p<0.005).

Conclusion: In patients with subacute and chronic lumbar discopathies, motorized traction and spinal decompression treatments added to conventional treatment were found to be more effective than conventional treatment alone. The results of spinal decompression and conventional motorized traction treatments appear to be similar.

## Introduction

Chronic low back pain is found in approximately 20% of individuals worldwide and it is a leading cause of disability [1]. Engaging in physically demanding jobs, having physical and mental comorbidities, smoking, and obesity are risk factors for low back pain [2]. Anatomical structures that have a potentially nociceptive contribution to low back pain are intervertebral discs, facet joints, and vertebral endplates [2].

Treatment modalities such as superficial and deep heating, massage, electrotherapy, and traction are used in the physical therapy of low back pain [3]. It has been determined that these treatments are effective in reducing pain and improving function in patients with lumbar disc herniation. However, the level of evidence for these modalities is low to moderate. Despite this, exercise and traction are recommended [4].

Different traction types have been reported in the literature, e.g., manual traction, mechanical or motorized traction, auto traction, and underwater traction. Manual traction is most commonly used in clinical practice, followed by motorized traction. Traction can be continuous (continuing up to 20 minutes or longer) or intermittent (alternating traction and cyclic relaxation with several minutes) depending on the application; also, strength, rhythm, and patient positioning can vary [5]. Variability in how treatments are applied could affect outcomes and make it challenging to compare effectiveness directly. It has been suggested that traction reduces the compressive forces on the discs by separating the vertebral bodies, reduces nerve root compression by expanding the intervertebral foramen, and helps restore herniated discs by creating tension in the spinal ligaments [6]. Horizontal traction has been shown to be significantly effective in increasing the disc height at the lower lumbar levels, especially in the posterior regions of the discs [7].

A relatively new technique, non-surgical spinal decompression, uses a sensitive computerized mechanism and expands spinal nerve roots by segmental distraction [8]. The basis of the mechanism of action of spinal decompression is the reduction of intradiscal pressure. This facilitates oxygen and nutrient uptake and increases the metabolism and healing of the disc [9]. Although spinal decompression devices theoretically allow the distraction of the spine without reactive muscle contraction and allow the vertebrae to separate better, there is little evidence that they differ from simple traction because it is unclear whether muscle tension plays a significant role in altering vertebral displacement during traction [10].

There is a limited number of studies comparing non-surgical spinal decompression with other treatment options such as conventional motor traction in the literature because of the difficulty in designing appropriate trials [11]. In this retrospective study, we aimed to compare the effectiveness of these treatments, which are utilized routinely in our clinic.

This article was previously posted to the Research Square preprint server on March 09, 2023.

## Materials and methods

Participants

The records of patients aged 20-65 years who underwent physical therapy to treat lumbar intervertebral disc disorders (with ICD codes M51 and its subcodes) in Afyonkarahisar Health Sciences University, Department of Physical Medicine and Rehabilitation, between 1 January 2019 and 10 November 2022 were reviewed. The records of routine anamneses, examinations, treatment cards, and control evaluation records of the patients in the outpatient clinic were retrieved from our hospital’s electronic database. Patients with acute low back pain (<6 weeks), history of lumbar surgery, spondylolisthesis or spinal stenosis, inflammatory rheumatic disease, sequestered hernia, patients receiving treatment other than the defined standard conventional physiotherapy (eg, massage therapy, balneotherapy, hydrotherapy, etc.), patients with less than 10 sessions of treatment, and those for whom sufficient data could not be obtained in the records were excluded.

The patients were divided into three groups according to the treatments they received. The first group consisted of patients who received conventional therapy alone, the second group consisted of patients who received motorized traction therapy in addition to conventional physiotherapy, and the third group consisted of patients who received non-surgical spinal decompression therapy in addition to conventional therapy.

Conventional Physiotherapy Group

In conventional physiotherapy, lumbar joint range of motion and core stabilization exercises were carried out. Additionally, transcutaneous electrical nerve stimulation (TENS) as an analgesic electrotherapy method (Chattanoga Intelect Advanced 2773MS device), ultrasound as a deep heating device (Chattanoga Intelect mobile model 2776), and a hot pack as a superficial heating device was applied. TENS was applied in conventional mode for 20 minutes with two sets of 40 x 40 mm electrodes placed in the lumbar region. Ultrasound was applied to the lumbar region paravertebral muscles for 10 minutes with 1.5 W/cm2 intensity and 1 MHZ frequency in continuous mode. Superficial hot pack treatment was applied for 20 minutes with a towel covered around the hot pack, in a way that would not disturb the patient. Patients performed lumbar range of motion and core stabilization exercises (quadruped exercise, prone plank, side plank, superman, etc.) for 20 minutes in every therapy session under the supervision of trained physiotherapists with at least five years of experience. Data from patients who underwent different physiotherapy protocols other than this combination were excluded from the study.

Motorized Traction Group

**Figure 1:**
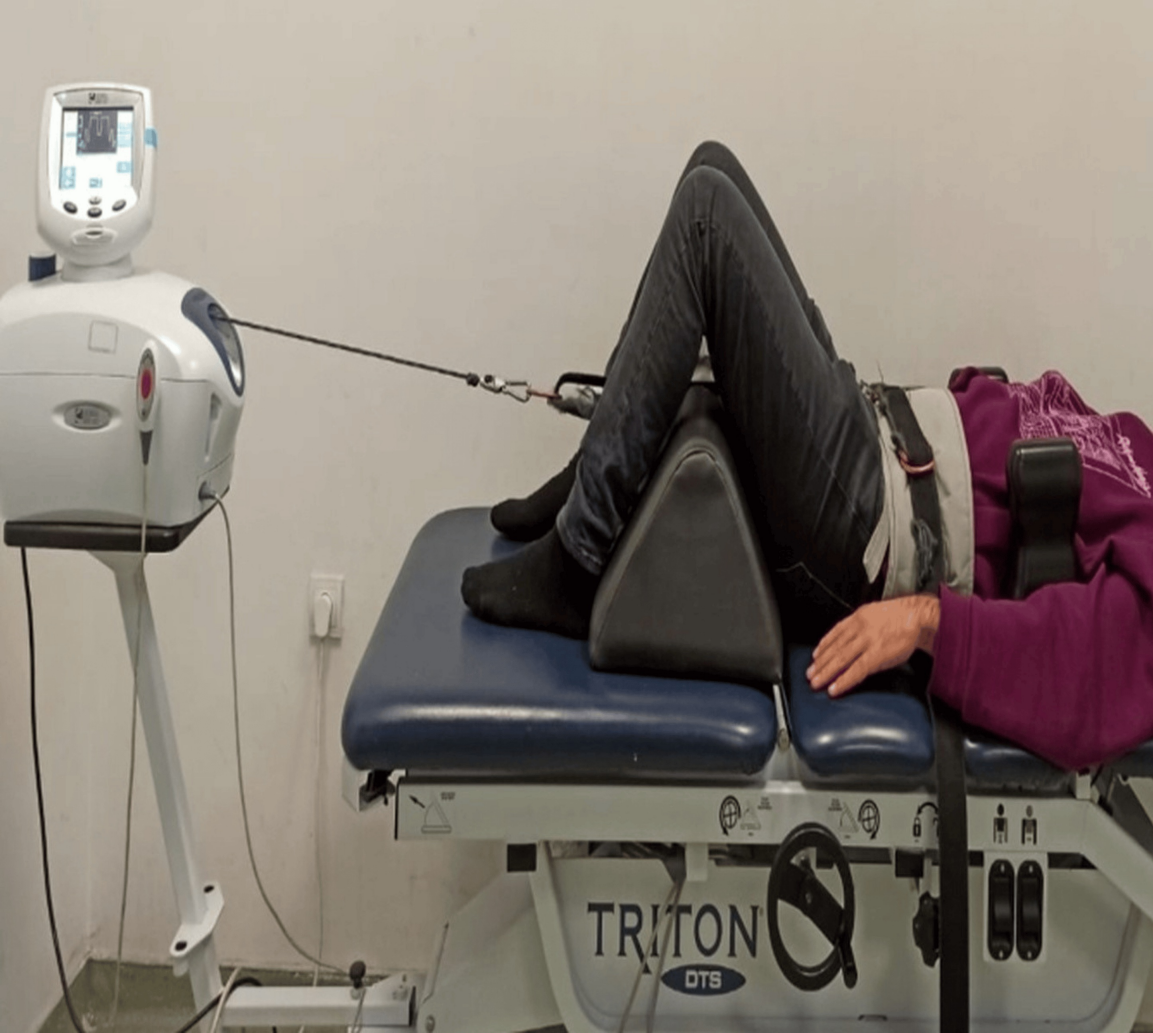
Conventional motorized traction application

Patients who received motorized traction treatment with the Chattanooga Triton Traction Machine 4798 for three days a week in addition to conventional physiotherapy were included in this group. The patient was positioned on the traction bed in the supine position with the hips and knees flexed. The traction belt was attached around the pelvis (Figure 1). Traction was applied for 20 minutes. Intermittent traction was applied in three steps: a 10-second (sec) pull and 10-second release in the first step, a 60-second pull, a 20-second release in the second step, and a 10-second pull and 10-second release in the third step. The traction and release forces were adjusted to be half and one-fourth of the patient's body weight, respectively. The patient was provided a safety switch button, so when the switch button is pressed (if the patient feels any discomfort or unusual pain) the device stops traction.

Spinal Decompression Group

**Figure 2:**
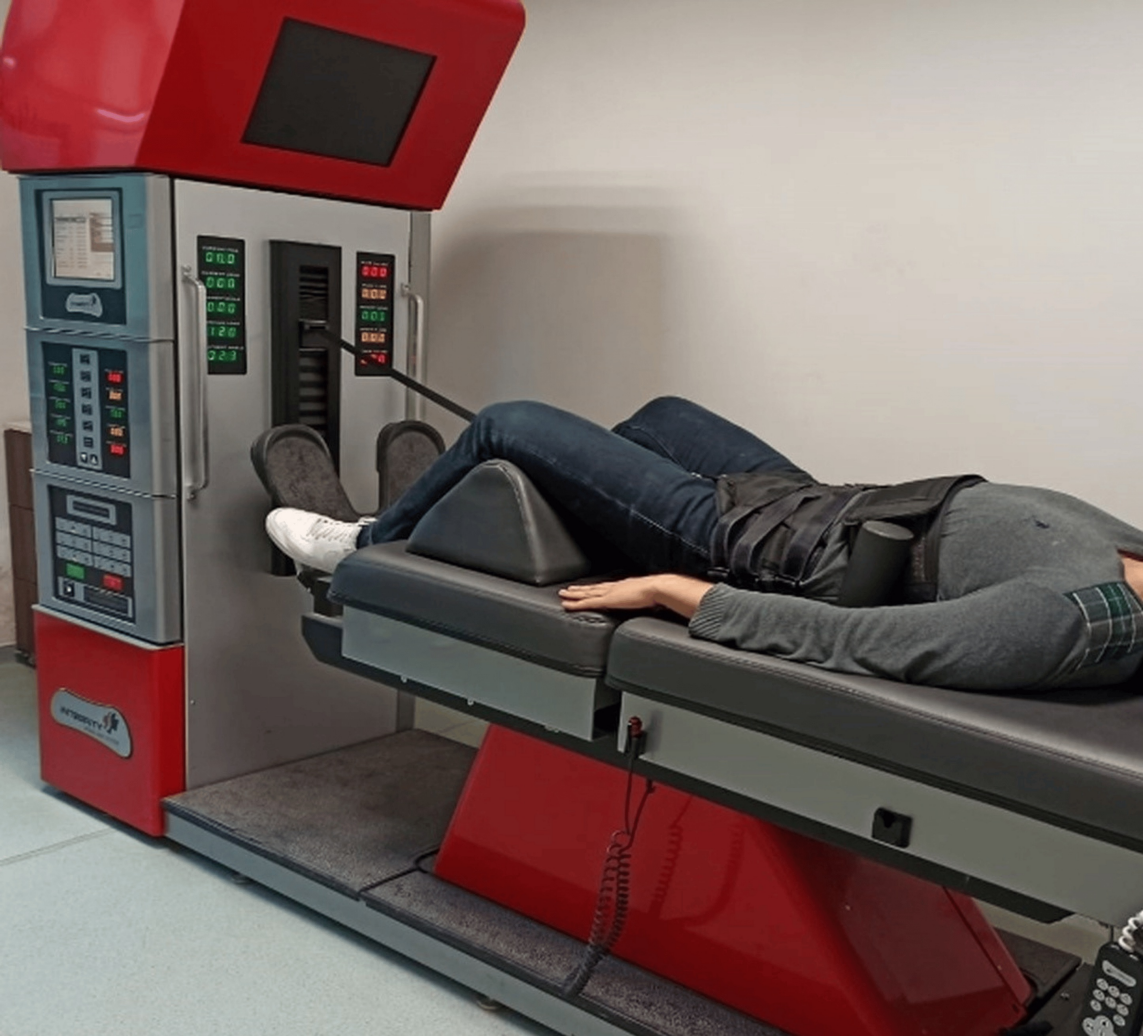
Non-surgical spinal decompression application

Patients who received spinal decompression therapy with the ISCS 2.0 Integrity Spinal Care System device three days a week in addition to conventional physiotherapy were included in this group. The patients were positioned in the supine position supported by the table with their knees in flexion. They were fastened to the table with three belts, the first on the chest, the second under the rib cage, and the third on the iliac wing (Figure 2). Decompression started with 10 lbs (4.54 kg), less than half of the patient's weight, and was increased to half of their weight in the second session as specified in the device application protocol to ensure patient tolerance. Since it is stated in the literature that effective traction force is possible with half of the body weight, in the following sessions, the traction force was applied in a standard way, 10 lbs more than half of the body weight. Each session lasted approximately 20 minutes. The relaxation was continued until the pulling force reached half of the patient's weight; when it reached this level, traction was applied by the device again. The relaxation time was set at 30 seconds and the withdrawal time at 60 seconds. In this process, the traction angle was set to 30 degrees. The patient was given a safety switch button, so when the switch button was pressed (if the patient felt any discomfort or unusual pain) the device stopped traction. In addition, to ensure safety, the physiotherapist supervising the treatment monitored the patient, and the treatment was terminated in the presence of pain or any disturbing symptoms in the patient.

Evaluation parameters

Demographic data of the patients, symptom durations, medical treatments, examination findings, lumbosacral MRI findings, method and duration of the treatment given, and the results of VAS and ODI before/after the treatment were recorded.

Visual Analogue Scale

The pain VAS is a scale consisting of a horizontal or vertical line, usually 10 centimeters (100 mm) long. For pain intensity, the scale is fixed at "no pain" (0 points) and "worst possible pain" or "worst pain imaginable" (100 points [10 cm scale]) [12].

Oswestry Disability Index

ODI has 10 questions about activities of daily life that are affected by low back pain, such as standing, walking, weightlifting, sitting, dressing, traveling, sleeping, and self-care. Each item is scored from 0 to 5, with higher values ​​representing greater disability. The total score is multiplied by 2 and expressed as a percentage. Its reliability and validity have also been demonstrated in Turkish populations [13].

Statistical analysis

The statistical analysis was performed using IBM SPSS v. 20.0 (IBM Corp., Armonk, NY). The distribution of continuous variables was evaluated using the Shapiro-Wilk test. For descriptive statistics, the number of units (n), percentage (%), and median (range) values were given. For comparisons of two independent groups using non-parametric data, the Mann-Whitney U-test was performed. For comparisons of three independent groups using non-parametric data, the Kruskall-Wallis test was used. P values <0.05 were considered significant. Significance values have been adjusted by the Bonferroni correction for multiple tests.

## Results

A total of 160 patients were evaluated. The mean age of the patients was 44.6±12.4 years (range 21-65), 57.5% (n=92) were female and 42.5% (n=68) were male. Of the patients, 87.5% (n=140) were using nonsteroidal anti-inflammatory drugs, 11.9% (n=19) were using gabapentin/pregabalin and 0.6% (n=1) were using opiate analgesics. The rate of patients who received conventional physiotherapy alone was 49.4% (n=79), for those who received motorized traction was 22.5% (n=36), and who received spinal decompression was 28.1% (n=45).

Comparisons of gender, occupation, clinical findings and MRI findings by group are given in Table 1. The decompression level of 62.2% of patients who underwent spinal decompression was L5-S1, 35.6% was L4-L5 and 2.2% was L3-4.

**Table 1:** Comparison of demographic data and percentage statistics of clinical characteristics of the groups p^a^ = Statistical significance level of the difference between conventional physiotherapy and traction groups, chi-square test p^b^ = Statistical significance level of the difference between conventional physiotherapy and spinal decompression groups, chi-square test p^c^ = Statistical significance level of the difference between traction and spinal decompression groups, chi-square test MRI: Magnetic resonance imaging

Variables		Conventional physiotherapy; n=79 (%)	Conventional motorized traction; n=36 (%)	Non-surgical spinal decompression; n=45 (%)	p^a^	p^b^	p^c^
Sex	Male	41.8	41.7	44.4	0.992	0.772	0.802
	Female	58.2	58.3	55.6			
Hard work	Yes	35.4	19.4	51.1	0.084	0.088	0.003
	No	64.6	80.6	48.9			
Straight leg raise test	Positive	62	36.1	57.8	0.010	0.642	0.052
	Negative	38	63.9	42.2			
Neurological deficit	Yes	7.6	11.1	6.7	0.535	0.848	0.479
	No	92.4	88.9	93.3			
Hernia level	Single	20.3	5.6	48.9	0.044	0.001	<0.001
	Multilevel	79.7	94.4	51.1			
MRI findings	Bulging	54.4	50	15.6	0.659	<0.001	<0.001
	Hernia	45.6	50	84.4			

There was no difference between the conventional physiotherapy, motorized traction, and spinal decompression groups in terms of age, duration of symptoms, and the number of sessions (p>0.05). In both three groups, the mean VAS and ODI scores were significantly decreased in the pre-and post-treatment comparisons (p<0.005) (Table 2).

The initial VAS and ODI values ​​of the groups were not similar; therefore the changes in percentage rates of VAS and ODI were compared. Change rates in VAS scores were higher in the traction group and spinal decompression group than in the conventional treatment group (p<0.001, p=0.019, respectively). There was no difference between the traction and spinal decompression groups in terms of VAS change rates (p=0.081). ODI change rates were higher in the traction group and spinal decompression group than in the conventional treatment group (p=0.001, p=0.025, respectively). There was no difference between the traction and spinal decompression groups in terms of ODI change rates (p=0.291) (Table 2).

**Table 2:** Comparison of age, symptom duration, number of sessions, and VAS, ODI values of the groups p : Statistical significance level of the difference between three groups, Kruskall-Wallis test. a-b: There is no difference between groups with the same letter for each row. Significance values have been adjusted by the Bonferroni correction for multiple tests. p*: Intra-group comparisons of values before and after treatment, Kruskall-Wallis test. VAS BT: visual analog scale mean values before treatment VAS AT: visual analog scale mean values after treatment VAS improvement: percentage change rate of VAS after treatment ODI BT: Oswestry Disability Index mean values before treatment ODI AT: Oswestry Disability Index mean values after treatment ODI improvement: percentage change rate of ODI after treatment Mean ± SD: mean ± standard deviation

Variable Mean ± SD	Conventional physiotherapy; n=79	Conventional motorized traction; n=36	Non-surgical spinal decompression; n=45	Test statistics	p
Age (years)	46.45±12.25	43.08±12.55	42.44±12.31	3.871	0.144
Symptom duration (months)	19.27±27.66	13.00±22.46	19.21±29.41	4.859	0.088
Number of sessions	15.54±1.54	16.20±2	16.17±2.79	2.216	0.330
VAS BT	6.01±1.66^a^	7.08±1.61^b^	6.2±1.58^a^	10.943	0.004
VAS AT	4.98±1.69	4.94±1.63	4.66±1.93	0.950	0.622
VAS BT-AT p*	<0.001	<0.001	<0.001		
VAS improvement (%)	16.63±18.38^a^	26.80±13.57^b^	22.44±19.81^b^	17.952	0.001
ODI BT	37.24±17.22	41.72±15.09	40.02±15.25	4.119	0.128
ODI AT	27.18±16.19	22.83±11.06	25.44±17.55	1.012	0.603
ODI BT-AT p*	<0.001	<0.001	<0.001	12.132	
ODI İmprovement (%)	19.73±66.94^a^	47.47±20.24^b^	37.28±45.52^b^		0.002

In the subgroup analyses of SLR-positive patients change rates in VAS scores were higher in the traction group and spinal decompression group than in the conventional treatment group (p=0.004, p=0.006 respectively). There was no difference between the traction and spinal decompression groups in terms of VAS change rates (p=0.648). ODI change rates were higher in the traction group and spinal decompression group than in the conventional treatment group (p=0.036, p<0.023, respectively). There was no difference between the traction and spinal decompression groups in terms of ODI change rates (p=0.553) (Table 3).

**Table 3:** Comparisons of groups in patients with positive straight leg raise test p : Statistical significance level of the difference between three groups, Kruskall-Wallis test. a-b: There is no difference between groups with the same letter for each row. Significance values have been adjusted by the Bonferroni correction for multiple tests. VAS BT: visual analog scale mean values before treatment VAS AT: visual analog scale mean values after treatment VAS improvement: percentage change rate of VAS after treatment ODI BT: Oswestry Disability Index mean values before treatment ODI AT: Oswestry Disability Index mean values after treatment ODI improvement: percentage change rate of ODI after treatment Mean ± SD: mean ± standard deviation

Variable Mean±SD	Conventional physiotherapy; n=49	Conventional motorized traction; n=13	Non-surgical spinal decompression; n=26	Test statistics	p
Age (years)	45.36±11.60	45.61±12.08	42.80±12.22	1.016	0.602
Symptom duration (months)	23.24±32.53	4.69±3.37	24.57±36.03	3.475	0.176
Number of sessions	15.57±1.56	15.38±1.38	16.19±3.05	1.255	0.534
VAS BT	5.91±1.61	6.84±1.99	6.38±1.94	4.927	0.085
VAS AT	5.1±1.68	5.07±1.89	4.61±2.28	0.752	0.687
VAS improvement (%)	13.25±16.01^a^	27.21±15.33^b^	30.11±27.3^b^	12.710	0.002
ODI BT	39.16±19.01	38.46±17.81	40.88±16.02	0.241	0.887
ODI AT	30.46±17.44	23.69±13.63	26.5±18.08	1.187	0.552
ODI improvement (%)	10.75±80.41^a^	45.84±24.86^b^	33.58±57.14^b^	7.533	0.023

## Discussion

The current study aimed to compare the effectiveness of conventional physiotherapy and motorized traction with spinal decompression treatments added to conventional physiotherapy in patients with low back pain. It was found that motorized traction and spinal decompression treatments added to conventional physiotherapy were more effective for pain and disability compared to conventional physiotherapy alone.

Lumbar traction therapy refers to any method of distracting the lumbar vertebrae with force directed along the lower-upper axis of the spine to treat chronic low back pain. It became a common alternative for the treatment of chronic low back pain in the early 20th century, and theories were developed on how traction should be applied, including discussions about the ideal amount of force, duration of traction, and timing of force intervals [10]. Cyriax stated that traction could be used not only for chronic low back pain, but also for lumbar disc lesions, and he theorized that traction would create negative pressure on the disc and thus reduce disc herniations [10]. In recent years, several imaging studies have yielded findings that support this theory. Liu et al. evaluated the water content in the intervertebral disc after traction by MRI in patients with chronic low back pain and investigated the relationship between MRI changes and the ODI and VAS score. In each patient, the mean MRI changes in the nucleus pulposus of the five discs and the changes in the ODI/VAS scores showed a strong correlation. While this study showed the clinical and anatomical effects of traction, it also provided evidence about the traction mechanism of action [14].

Although motorized traction devices are capable of producing specific measurable forces and rhythms, most studies do not have any standardized traction protocols [15]. Especially the number of sessions and traction force differ in protocols. In a study in which traction forces of one-fifth, one-third, and half their body weight were applied, all three forces were equally effective in immediately improving the straight leg raise test (SLR) angle in patients with lumbar protruded intervertebral discs; however, improvement in low back pain was observed only in the group that traction was with half the body weight [16]. Traction therapy with both 30% and 60% of body weight has been shown to be effective in improving the angle of the SLR test [17]. As a result, the recommended amount of force for traction varies, however, it is considered that traction performed with 30-50% of body weight is the most effective [10]. In light of this data, a traction protocol with half of the body weight is generally applied in our clinic. In our study, we evaluated the results of patients who underwent intermittent traction with half of their body weight for 20 minutes. According to the results of our study, we can say that similar to the literature, traction with half of the body weight is effective on pain and disability.

According to the result of a systematic review, there is low- to moderate-quality evidence for people with mixed symptom patterns [18]. The systematic reviews and guidelines are mostly based on research using heterogeneous subjects with nonspecific low back pain. Clinical experts advocate the use of mechanical traction for more specific patient sub-groups with low back pain, particularly with sciatic and nerve root compression symptoms [19]. In a study performed with 64 patients with low back and leg pain and symptoms of nerve root compression, two groups were compared to receive an exercise intervention with or without mechanical traction and the results of this study suggested that traction may be more effective in the presence of leg symptoms, signs of nerve root compression and in patients characterized by crossed straight leg raising [19]. In our study, in the subgroup analysis formed of SLR test-positive patients, we found the effects of motorized traction and spinal decompression added to conventional physiotherapy to be superior to conventional physiotherapy alone, regarding pain and disability.

In our study, it was observed that the rates of those who worked in physically strenuous jobs were higher in the patient group with spinal decompression. We thought that it could be related to the fact that physicians prefer treatments that they think are more effective considering the patient's social history. This situation may have caused heterogeneity in the patient distribution in this retrospective study, therefore further randomized controlled studies are needed. Besides, it was seen that single-level hernia rates were higher in the spinal decompression group. In this method, an effective traction force can be applied to a specific disc without leading to paraspinal muscle spasm reflexes [9]. Therefore, spinal decompression therapy could have been preferred more in patients with a significant single-level hernia. There are a limited number of studies showing that spinal decompression treatment provides an improvement in MRI findings in addition to its clinical effect in patients with subacute and chronic low back pain [20,21]. Apfel et al. stated that there is a relationship between the restoration of disc height and pain relief [20].

Besides the studies examining the effect of spinal decompression alone, the effectiveness of treatments in which spinal decompression combined with conventional methods was also evaluated. As an example, Demirel et al. stated that both combination treatment and conventional treatment alone had positive effects on pain relief, functional restoration, and reduction of hernia thickness. In addition, they reported that although the reduction in hernia size was more in the group in which spinal decompression was added compared to the conventional treatment, they did not find a difference between the groups [22]. However, in our study, we found that combined therapy was more effective on pain and disability. This difference in results may be due to the difference in the conventional treatment methods applied in each study, as well as the difference in the patient groups. For example, in the study of Demirel et al., deep friction massage was used as a conventional method in addition to exercise [22]. In our study, different from Demirel et al.'s study, hot packs, TENS, Ultrasound, and exercise protocols were used. Also, different from this study, we also evaluated patients with multiple hernias. Concurrent with our study, Amjad et al. reported that in patients with subacute lumbar radiculopathy, combination treatment was more effective in relieving pain than conventional physical therapy alone, in which they applied spinal decompression therapy in addition to routine conventional physical therapy [8].

There are a limited number of studies in the literature comparing non-surgical spinal decompression with conventional motorized traction. The study of Koçak et al. showed that both conventional motorized traction and spinal decompression are effective methods in pain management to improve the functional status and depressive moods in patients with chronic lumbar disc herniation, and that non-surgical spinal decompression was not superior to conventional motorized traction in terms of pain, functionality, depression, and quality of life [11]. In a review evaluating the effectiveness of motorized traction and nonsurgical spinal decompression for chronic discogenic lumbosacral pain, six of seven randomized studies reported no difference between motorized traction and spinal decompression [9]. In our study, we also found the two groups were similar. The strength of our study is that it is the first study to compare spinal decompression and traction added to conventional treatment, both with each other and with conventional treatment alone. Also, the evaluation of patients with heterogeneous examination and MRI findings and the subgroup with positive SLR strengthens the results of our study.

Limitations

Due to the retrospective nature of our study, the heterogeneity of the patient groups and the small number of patients are limitations. In addition, the fact that factors such as body mass index, accompanying fibromyalgia, drug use, different decompression levels, exercise compliance, lifestyle factors, or psychological factors (e.g., fear-avoidance beliefs, anxiety about pain), which have the potential to affect the effectiveness of treatments and results were not evaluated may also have affected our results. The exclusion criteria might limit the generalizability of the study to only patients with specific characteristics, reducing the diversity of the sample. Due to these limitations, it has insufficient results in terms of clinical acceptance. Randomized controlled trials with balanced group sizes would provide more reliable comparisons between the treatments.

## Conclusions

According to the results of our study, motorized traction and spinal decompression treatments added to conventional treatment in patients with subacute and chronic lumbar discopathy appear to be effective, although the retrospective nature and design of the study limit the generalizability of this result. More prospective randomized controlled trials comparing the relatively new spinal decompression treatment with conventional traction are needed.
