# Correction: Comparison of the Effectiveness of Traditional Motorized Traction and Non-surgical Spinal Decompression Therapy Added to Conventional Physiotherapy for Treatment of Chronic Low Back Pain

**DOI:** 10.7759/cureus.c193

**Published:** 2024-09-23

**Authors:** Sevda Adar, Onurhan Apaydın, Umit Dündar, Hasan Toktas, Hilal Yesil, Selma Eroglu, Nuran Eyvaz

**Affiliations:** 1 Physical Medicine and Rehabilitation, Afyonkarahisar Health Sciences University, Afyonkarahisar, TUR

This article has been corrected to replace an incorrect reference to DRX9000. The ISCS 2.0 Integrity Spinal Care System was used by the authors and has replaced the incorrect mention of the DRX9000 in the first sentence of the first paragraph under the subheader 'Spinal Decompression Group'. The authors and journal regret that this error was not identified and addressed prior to publication.

